# Adherence to the EAT-*Lancet* sustainable diet and ultra-processed food consumption: findings from a nationwide population-based study in Brazil

**DOI:** 10.1017/S1368980024001678

**Published:** 2024-10-04

**Authors:** Leandro Teixeira Cacau, Thays Nascimento Souza, Maria Laura da Costa Louzada, Dirce Maria Lobo Marchioni

**Affiliations:** 1 Department of Nutrition, School of Public Health, University of São Paulo, São Paulo, Brazil; 2 Center for Epidemiological Research in Nutrition and Health, University of São Paulo, São Paulo, Brazil

**Keywords:** EAT-*Lancet* diet, Sustainable diet, Ultra-processed foods, Nutritional epidemiology

## Abstract

**Objective:**

To evaluate the association between ultra-processed food consumption and adherence to the EAT-*Lancet* diet in a representative sample of the Brazilian population.

**Design:**

The study used data from the Brazilian National Dietary Survey 2017–2018 and employed linear regression models to evaluate the association between ultra-processed food consumption and adherence to the EAT-*Lancet* diet, as measured by the Nova food system and Planetary Health Diet Index (PHDI), respectively.

**Setting:**

Nationally representative sample of the Brazilian population.

**Participants:**

The study included 46 164 Brazilians ≥ 10 years old.

**Results:**

The average PHDI total score was 45·9 points (95 % CI 45·6, 46·1). The ultra-processed food consumption was, with dose-response, inversely associated with the adherence to the EAT-*Lancet* diet. The PHDI total score was 5·38 points lower (95 % CI –6·01, –4·75) in individuals in the highest quintile of consumption of ultra-processed foods, as compared to those in the first quintile. The PHDI score was also inversely associated with the share of processed culinary ingredients and processed foods and positively associated with the share of unprocessed or minimally processed foods.

**Conclusions:**

Our study showed an inverse relationship between the consumption of ultra-processed foods and the adherence to a healthy and sustainable diet.

Diets are intrinsically linked to human and planetary health^(1)^. Unbalanced and nutritionally poor diets, usually characterised by low amounts of fruits, vegetables, nuts and whole grain cereals and high in red and processed meat, are responsible for a major part of the global burden of diseases^(2)^, which is explained due to the food transition from traditional diets to globalised diets. Alongside the diet-disease scenario, the global food system is nowadays one of the primary drivers of adverse environmental impacts. Agriculture is responsible for about 30 % of all greenhouse gas emission (GHGE), uses 70 % of the freshwater resource and occupies about 40 % of the Earth’s surface, leading to an adverse environment and crossing the planetary boundaries^(3,4)^.

The Food and Agriculture Organization of the United Nations (FAO) defines sustainable healthy diets as those that enhance all aspects of individual health, exert minimal environmental pressure and are accessible, affordable, safe, equitable and culturally acceptable on a global scale^(5)^. Adherence to sustainable dietary patterns has been related to reduce diet-related mortality rates and environmental impacts, such as GHGE, water and land use^(3,4,6,7)^. Achieving a worldwide shift from unhealthy, umbalanced diets to healthy, sustainable ones is one of the most significant challenges of this century^(1)^.

To guide countries, researchers and policy makers, the EAT-*Lancet* Commission proposed a global sustainable reference diet that promotes human and planetary health. This model diet, called ‘planetary health diet’, primarily includes vegetables, fruits, whole grains, legumes, nuts and unsaturated oils, while suggesting only low–to-moderate amounts of seafood and poultry, and minimal to no red meat, animal fats, added sugar, refined grains and starchy vegetables^(8)^. On a global adoption scenario, this reference diet was related to reduce diet-related deaths by 11·6 million annually (i.e. 23·6 % deaths per year) and decrease GHGE by 42 %, freshwater use by 10 % and nitrogen application by 15 %^(7,8)^.

Although the EAT-*Lancet* report has been a landmark in the discussion of sustainable food systems for proposing a healthy and sustainable model diet, it has received some criticism for overlooking the negative effects of ultra-processed foods on human and planetary health^(9,10)^. Ultra-processed foods, as defined by the Nova food classification, are industrial formulations typically ready-to-eat, often derived from high-yield crops, such as sugars and syrups, refined starches, oils and fats, as well as protein isolates and low-commercial-value animal tissues. These formulations are designed to be visually appealing and have very intense flavours, achieved through combinations of flavourings, colourings, emulsifiers, sweeteners, thickeners and other cosmetic additives^(11)^. Several systematic reviews and meta-analyses showed the association between ultra-processed foods and poor-diet quality^(12–14)^, higher risk of non-communicable diseases and all-cause mortality^(15–19)^, biodiversity loss^(20)^, as well as the temporal relationship between increasing ultra-processed food consumption and increasing GHGE, water and land use^(21)^.

Despite the evidence that ultra-processed foods have negative impacts on human and planetary health, the EAT-*Lancet* Commission report overlooks this effect and does not use or cite food processing as something to be avoided. Therefore, we wonder if these two diet quality metrics (i.e. the share of ultra-processed foods and the EAT-*Lancet* diet), increasingly recognised in the literature, are associated, even though they originate from different theoretical assumptions. Currently, there is no evidence on this field. Hence, the aim of this study was to address this question in a nationally representative sample of the Brazilian population.

## Methods

### Study design

The 2017–2018 National Dietary Survey (NDS) was used for this analysis. The NDS was integrated into the Household Budget Survey (HBS), a nationally representative survey conducted by the Brazilian Institute of Geography and Statistics to measure household consumption, expenditures and income, thus providing a comprehensive profile of the living conditions of the Brazilian population^(22,23)^.

Briefly, the HBS employed a two-stage cluster sampling methods, randomly selecting census sector and households with socioeconomic and geographical stratifications of the primary sampling units based on the 2000 Demographic Census. Data collection occurred from July 2017 to July 2018. Overall, the 2017–2018 HBS included 57 920 households, of which a subsample of 20 112 participated in the NDS, totalling 46 164 individuals aged ≥10 years with data on individual food consumption. Further details on sampling and data collection are available in previous publications by the Brazilian Institute of Geography and Statistics^(22,23)^.

### Individual food consumption assessment

Dietary data were gathered using 24-hour dietary recalls conducted on two non-consecutive days at the respondent’s home by trained research agents. These interviews followed a structured approach based on the Automated Multiple-Pass Method, utilising specialised software on a tablet^(23)^.

Initially, participants were asked to list all foods and beverages consumed the previous day, creating a ‘quick list’. Following this, detailed information was recorded about the foods, beverages and recipes, including portion sizes in household measures, preparation and cooking techniques, added items, eating occasions and meal locations. The Table of Reference Measures for Food Consumed in Brazil was then used to convert the food and beverage quantities into grams and millilitres^(24)^.

To determine the energy and nutritional composition, the Brazilian Food Composition Table (TBCA-USP) version 7·0 was employed (https://tbca.net.br/indexen.html). The TBCA is a web-based resource comprising two databases: (1) the Nutrient Intake Evaluation Database, which offers the complete nutrient profile of Brazil’s most-consumed foods, and (2) the Biodiversity and Regional Foods Database, which centralises analytical data on the biodiversity of Brazilian foods and typical regional dishes. Information for the TBCA was obtained through direct analytical analyses of foods and by compiling data from previous publications, analytical reports from the food industry and other food composition tables. Detailed information regarding the TBCA can be found in other sources^(25,26)^. For this study, only the first 24-hour dietary recalls was used.

### Adherence to EAT-Lancet dietary recommendations

The Planetary Health Diet Index (PHDI) was used to evaluate adherence to the EAT-*Lancet* diet, which is a 16-component dietary index that considers all food groups proposed in the EAT-*Lancet* diet with a continuous score for each component. The PHDI total score was previously validated and performed satisfactorily in terms of validity and reliability criteria and was associated with lower GHGE and higher overall dietary quality^(27)^.

The PHDI total score was calculated through the procedure described by Cacau *et al.* ^(27)^ and Marchioni *et al.* ^(28)^. Briefly, we identified foods classified as unprocessed or minimally processed (such as plain cooked fruits and vegetables) and culinary preparations composed of multiple ingredients (like lasagne, stroganoff and cakes), which required disaggregating into ingredient components to classify them into PHDI categories. Culinary preparations were disaggregated based on standard homemade recipes found in national literature. For ultra-processed foods with specific main ingredients (e.g. savoury chips made from maize starch), we estimated energy distribution using added sugars and total fat levels from the nutrient database. For example, in savoury chips, the percentage of energy from total fat was assumed to represent the contribution of the vegetable oil fraction in the food. After subtracting the total fat from the savoury chips, the contribution of the refined grain group (such as corn starch) was assumed to represent the remaining energy value of the food. Processed meats, like sausage, ham and salami, were not disaggregated into individual ingredients but rather categorised based on their primary ingredient origin or commonly marketed formulation within the respective groups of red meats or chicken and substitutes. More information can be found in the original paper elsewhere^(27)^. After the disaggregation process, the ingredients were allocated in their respective PHDI component. Examples of foods and ingredients included in the PHDI components are described in Supplementary Table S1.

The 16 PHDI components are divided into four categories: (1) adequacy components (nuts and peanuts, fruits, vegetables, vegetables and whole grains), (2) optimal components (eggs, dairy, fish and seafood, tubers and potatoes, and vegetable oils), (3) ratio components (dark green vegetables/total vegetables and orange-red vegetables/total vegetables) and (4) moderation components (red meat, poultry and substitutes, animal fats and added sugars). Table 1 presents the PHDI components with their respective cut-off points and scoring criteria.

**Table 1:** The Planetary Health Diet Index (PHDI) components and criteria for scoring system (recommended and lower or upper limit recommended values*)

Components	Range of score	Recommended value (i.e. criteria for maximum score, 10 or 5 points)	Criteria for minimum score (i.e. zero points)	Lower or upper limit of recommended intake (i.e. cut-off points for gradual scoring)
**Adequacy components**				
Fruits	0–10	≥5 % of total EI	Zero	–
Vegetables	0–10	≥3·1 % of total EI	Zero	–
Nuts and peanuts	0–10	≥11·6 of total EI	Zero	–
Whole cereals	0–10	≥11·3 % of total EI	Zero	–
Legumes^†^	0–10	≥32·4 % of total EI	Zero	–
**Optimum components**				
Eggs	0–10	0·8 % of total EI	Zero or >1·5 % of total EI	>0·8 % to 1·5 % of total EI
Dairy^‡^	0–10	6·1 % of total EI	Zero or >12·2 % of total EI	>6·1 % to 12·2 % of total EI
Tuber and potatoes	0–10	1·6 % of total EI	Zero or >3·1 % of total EI	>1·6 % to 3·1 % of total EI
Vegetable oils^§^	0–10	16·5 % of total EI	Zero or >30·7 % of total EI	>16·5 % to 30·7 % of total EI
Fish and seafood	0–10	1·6 % of total EI	Zero or >5·7 % of total EI	>1·6 % to 5·7 % of total EI
**Ratio components**				
DGV/total vegetables^||^	0–5	29·5 %	Zero	100 %
ReV/total vegetables^¶^	0–5	38·5 %	Zero	100 %
**Moderation components**				
Red meat^**^	0–10	Zero	>2·4 % of total EI	>0 to 2·4 % of total EI
Chicken and substitutes	0–10	Zero	>5 % of total EI	>0 to 5 % of total EI
Animal fats^*†^	0–10	Zero	>1·4 % of total EI	>0 to 1·4 % of total EI
Added sugars	0–10	Zero	>4·8 % of total EI	>0 to 4·8 % of total EI
**PHDI total score**	0–150			

EI: energy intake.

* All values expressed as caloric ratios between each PHDI component and total daily energy intake.

† Legumes: including soy.

‡ Dairy: excluding dairy fats.

§ Vegetable oils: including palm oil.

|| Dark green vegetables (DGV)/total vegetables: ratio between the energy intake of dark green vegetables (numerator) and the total vegetable energy intake (denominator) multiplied by 100.

¶ Red and orange vegetables (ReV)/total vegetables: ratio between the energy intake of red and orange vegetables (numerator) and the total vegetable energy intake (denominator) multiplied by 100.

** Red meat: including beef, lamb and pork.

*† Animal fats: lard, tallow and dairy fats.

For the adequacy components, the maximum score (i.e. 10 points) was assigned if consumption met or exceeded the recommendation (i.e. the criteria for maximum score). Otherwise, the score was proportional, being calculated as follows:

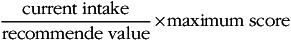




For example, subjects who consumed 3 % of energy from fruits from the total daily energy intake received 6 points = [(3 ÷ 5) × 10 = 6].

For the optimum components, the maximum score (i.e. 10 points) was assigned when reaching the recommended value. As consumption exceeds the recommended values, the score becomes inversely proportional to consumption, being calculated as follows:

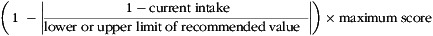




For example, subjects who consumed 0·4 % of energy from eggs of the total daily energy intake received 5 points [(1 – | 1 – 0·4 ÷ 0·8 |) × 10 = 5 points. For ratio components, the same logic as for optimum components was assigned.

Finally, the moderation components received a minimum score (i.e. 0 points) when consumption reached or exceeded the upper limit of recommended value (i.e. the criteria for minimum score). When consumption was lower than the consumption limit, the calculation was as follows:

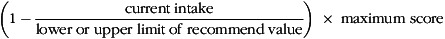




For example, subjects who consumed 1·5 % of energy from red meat of the total daily energy intake received 3·75 points [(1 – 1·5 ÷ 2·4) × 10 = 3·75].

Further detail on the PHDI development, scoring criteria, cut-off points and validity and reliability can be found elsewhere^(27,29)^.

### Ultra-processed food classification – Nova food system

Foods and beverages reported in the NDS 2017–2018 were also classified according to the Nova food system^(11)^.

Briefly, this classification was conducted by researchers who were properly trained and familiar with the Nova food system. The process involved listing all reported foods and beverages to identify single-ingredient and multi-ingredient items. Single-ingredient items consist of unprocessed or minimally processed foods (e.g. raw apple, milk and unsalted nuts) or processed culinary ingredients (e.g. salt and olive oil) and were directly included in the food list. Multi-ingredient items (e.g. pies, cakes, pasta dishes and sauces) were first categorised as culinary preparations or industrially manufactured products. Items identified as industrially manufactured (e.g. ready-made pie and frozen lasagne) were included in the food list as such. Those identified as culinary preparations (e.g. homemade pie and rice with broccoli) were disaggregated into their constituent ingredients using standard food recipes, with each ingredient listed separately. Subsequently, each item in the list was classified into one of the four Nova groups^(23,30)^.

The Nova food system includes four groups, namely (1) unprocessed or minimally processed foods, which are those consumed as obtained from the nature or that underwent industrial processes such as drying, boiling, freezing or others that do not add substances such as salt, sugar and/or oils or fats to the original food (e.g. fruits, vegetables, eggs and fresh meat); (2) processed culinary ingredients, which are substances obtained directly from nature or group 1 foods, which are used in the preparation, seasoning and cooking of foods (e.g. oils and fats, table sugar and salt); (3) processed foods, which are industrial products made by combining substances from group 2 with group 1 foods (e.g. cheese, fresh breads, vegetables in brine and salt-cured meat or fish); and (4) ultra-processed foods, defined as formulations of ingredients, mostly of exclusive industrial use, that result from several industrial processes and frequently are added by colours, flavours, emulsifiers and other cosmetic or sensory intensifying additives to make the final product palatable or more appealing (e.g. confectionary, salty snacks, sugary breakfast-cereal, packaged bread, carbonated soft drinks, flavoured yogurts, cookies and sausages). Thereafter, the share of contribution of each group to the total energy intake was estimated, i.e. the % energy intake of each Nova food group.

### Data analyses

The mean and the corresponding 95 % CI for PHDI total score and the % energy intake from each Nova food group were presented for the entire Brazilian population. Additionally, these data were stratified by sex (male, female), age group (<19, 19–30, 31–45, 46–59, or ≥60), per capita income (quartiles), self-reported race (white, black, brown, and Asian or Indigenous) and area of residence (urban or rural).

The association between the % energy intake of each Nova food group and the PHDI scores was first assessed using linear splines graphics. Subsequently, the population was stratified into quintiles of the % energy intake of ultra-processed foods, with the lowest consumers belonging to the first quintile and the highest consumers to the fifth.

Linear regression models were fitted to evaluate if the higher % energy intake of ultra-processed foods is associated with lower PHDI total score. In these models, the PHDI total score was the outcome variable, and quintiles of share of ultra-processed food group to total diet were the explanatory variable. The model was adjusted for sex (male and female), age group (<19, 19–30, 31–45, 46–59 or ≥60), per capita income (quartiles), self-reported race (white, black, brown, yellow/Asiatic, or indigenous) and residence area (urban or rural). Analyses of association were repeated considering the quintiles of the % energy intake of the non-ultra-processed food groups (i.e. unprocessed or minimally processed foods; culinary processed ingredients; and processed foods) with the same adjustments of covariables.

As a sensitivity analysis, linear regression models were fitted using the Nova food groups expressed as energy intake (kcal), rather than % energy intake. The models were adjusted for the same covariates as in the main analyses.

Data analysis was carried out on Stata® (Statistical Software for Professionals, College Station, Texas, USA), version 14·2 and using the survey module to consider the effect of complex sampling procedures adopted in the NDS 2017–2018 and in order to allow extrapolation of results for the Brazilian population. Statistically significant differences were considered when the 95 % CI did not overlap.

## Results

In the entire Brazilian population, the total PHDI had an average of 45·9 points (95 % CI 45·6, 46·1) and had a range of 1·65–95·0 points (i.e. minimum and maximum values). Higher PHDI scores were observed in women, in those with 31 years or older (31–45, 46–56 and ≥60 years), in individuals with higher per capita income and in those living in the urban area (Table 2).

**Table 2:** Distribution of the Planetary Health Diet Index (PHDI) and the caloric share (%) of Nova food groups for the Brazilian population and across sociodemographic characteristics (*n* 46 164). National Dietary Survey, 2017–2018

			Nova food groups							
	PHDI		Unprocessed or minimally processed foods		Processed culinary ingredients		Processed foods		Ultra-processed foods	
	Means	95 % CI	Means	95 % CI	Means	95 % CI	Means	95 % CI	Means	95 % CI
All	45·9	45·6, 46·1	53·4	53·0, 53·8	15·6	15·4, 15·8	11·3	11·1, 11·5	19·7	19·3, 20·1
Gender										
Male	45·4	45·2, 45·7	54·1	53·6, 54·6	15·0	14·7, 15·2	11·8	11·5, 12·1	19·1	18·6, 19·6
Female	46·3	46·0, 46·6	52·8	52·3, 53·2	16·2	15·9, 16,4	10·8	10·5, 11·1	20·3	19·8, 20·8
Age group (years)										
<19	43·6	43·2, 44·1	49·2	48·3, 50·1	14·0	13·6, 14·3	10·1	9·7, 10·6	26·7	25·6, 27·7
19–30	44·4	43·9, 44·9	51·5	50·8, 52·2	14·9	14·5, 15·3	10·8	10·4, 11·3	22·8	22·0, 23·6
31–45	45·9	45·5, 46·2	53·9	53·2, 54·6	16·3	16·0, 16·6	11·3	10·9, 11·7	18·5	17·8, 19·2
46–59	47·1	46·7, 47·5	55·0	54·4, 55·6	16·2	15·9, 16·6	12·1	11·7, 12·5	16·7	16·1, 17·3
≥60	48·2	47·8, 48·7	56·9	56·2, 57·5	16·1	15·7, 16·5	12·0	11·5, 12·4	15·1	14·5, 15·6
Per capita income										
1st quartile	44·5	44·0, 44·9	57·9	57·1, 58·6	15·0	14·7, 15·4	10·8	10·3, 11·3	16·3	15·7, 16·9
2nd quartile	45·5	45·0, 45·9	54·5	53·8, 55·2	15·8	15·4, 16·1	11·3	10·8, 11·7	18·5	17·8, 19·2
3rd quartile	46·4	45·9, 46·9	52·6	51·6, 53·5	15·8	15·4, 16·2	11·1	10·7, 11·6	20·6	19·4, 21·6
4th quartile	46·8	46·3, 47·3	50·1	49·5, 50·7	15·7	15·3, 16·1	11·8	11·4, 12·3	22·4	21·7, 23·0
Self-reported race/skin colour										
White	46·1	45·8, 46·5	51·1	50·1, 51·6	15·8	15·5, 16·1	11·7	11·4, 12·1	21·4	20·9, 22·0
Black	46·2	45·6, 46·8	55·1	54·0, 56·2	15·5	15·0, 16·0	11·0	10·4, 11·7	18·4	17·4, 19·4
Brown	45·6	45·2, 45·9	55·3	54·7, 55·8	15·4	15·2, 15·7	11·0	10·7, 11·3	18·3	17·7, 18·9
Yellow /Asiatic	49·6	46·1, 53·0	51·9	48·8, 55·1	15·5	12·6, 18·3	7·8	5·6, 10·0	24·8	19·7, 29·8
Indigenous	41·3	39·3, 43·3	52·7	48·9, 56·4	16·6	14·4, 18·8	10·6	8·1, 13·1	20·1	16·0, 24·3
Residence area										
Urban	46·0	45·7, 46·3	52·0	51·1, 52·4	15·5	15·3, 15·7	11·8	11·5, 12·1	20·7	20·2, 21·2
Rural	45·1	44·7, 45·5	61·9	61·1, 62·6	16·2	15·8, 16·5	8·3	7·8, 8·7	13·7	13·1, 14·3

Values are means with their respective 95 % CI.

For the entire Brazilian population, around 20 % of the daily energy intake came from ultra-processed foods (19·7; 95 % CI 19·3, 20·1), while more than half came from unprocessed or minimally processed foods (53·4; 95 % CI 53·0, 53·8). Processed culinary ingredients corresponded to around 16 % (15·6; 95 % CI 15·4, 15·8) and processed foods to nearly 11 % (11·3; 95 % CI 11·1, 11·5). The % energy intake of the subgroups of the four Nova food groups is described Supplementary Table S2.

Ultra-processed food consumption was slightly higher among females and adolescents (those with <19 years), but tended to decrease progressively with age, and was lower in those with ≥60 years. Furthermore, ultra-processed food consumption was higher in individuals with higher per capita income and in those living in urban areas. On the other hand, unprocessed or minimally processed food consumption was slightly higher among males and in individuals with ≥60 years. Regarding self-reported race/skin colour, individuals who self-reported as black and brown had higher unprocessed or minimally processed foods intake (Table 2).

**Fig. 1:**
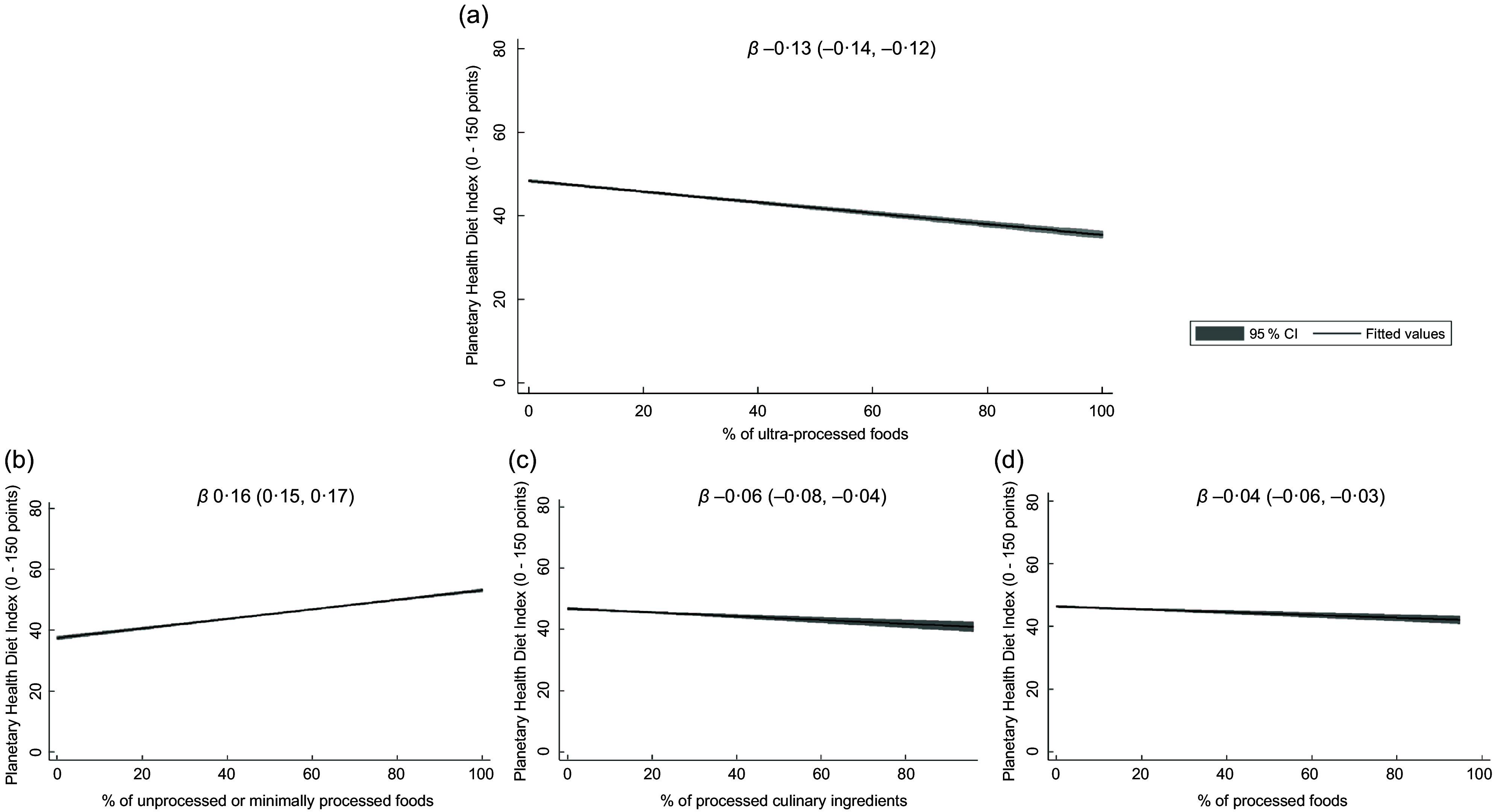
Crude linear splines of the association between the Planetary Health Diet Index (PHDI) score and the caloric share (%) of each Nova food group continuously (*n* 46 164). National Dietary Survey, 2017–2018. *β* coefficients and their respective 95 % CI. Legend: (a): linear spline of the association between the PHDI score and the caloric share (%) of ultra-processed foods. (b): Linear spline of the association between the PHDI score and the caloric share (%) of unprocessed or minimally processed foods. (c): Linear spline of the association between the PHDI score and the caloric share (%) of processed culinary ingredients. (d): Linear splines of the association between the PHDI score and the caloric share (%) of processed foods

Linear splines showed an inverse association between the % energy intake of ultra-processed foods and PHDI total score, as reported on the linear coefficients and their respective 95 % CI (–0·13; –0·14, –0·12) in Fig. 1(a). Associations in the same direction were observed for the processed culinary ingredients (Fig. 1(c)) and processed foods (Fig. 1(d)), while the % energy intake of unprocessed or minimally processed foods was directly associated with the PHDI total score (Fig. 1(b)).

**Fig. 2:**
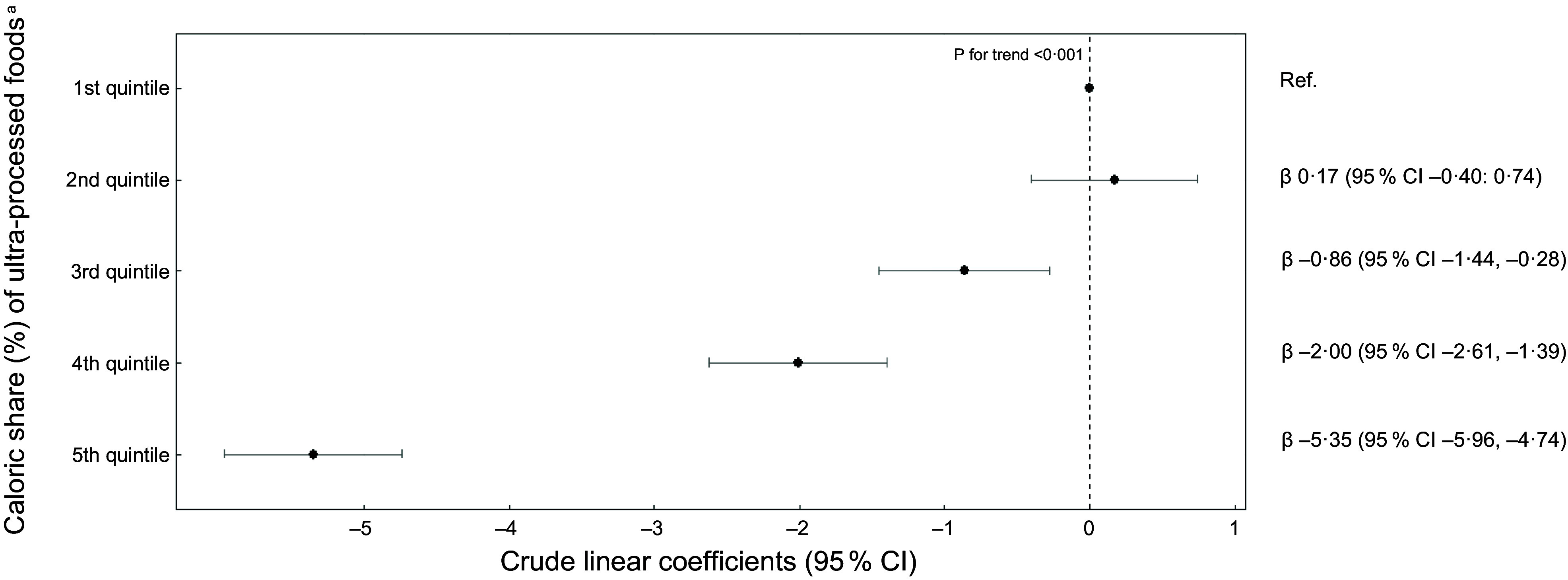
Crude linear coefficients and 95 % CI of the association between Planetary Health Diet Index (PHDI) scores and quintiles of the caloric share (%) of ultra-processed foods (*n* 46 164). National Dietary Survey, 2017–2018. Legend: ^a^ 1^st^ quintile: mean (min – max): 0·43 (0 – 3·40); 2^nd^ quintile 6·83 (3·40 – 9·92); 3^rd^ quintile 13·3 (9·92 – 17·3); 4^th^ quintile 22·8 (17·3 – 29·6); 5^th^ quintile 45·1 (29·6 – 100). Values are expressed as linear coefficients and 95 % CI

**Fig. 3:**
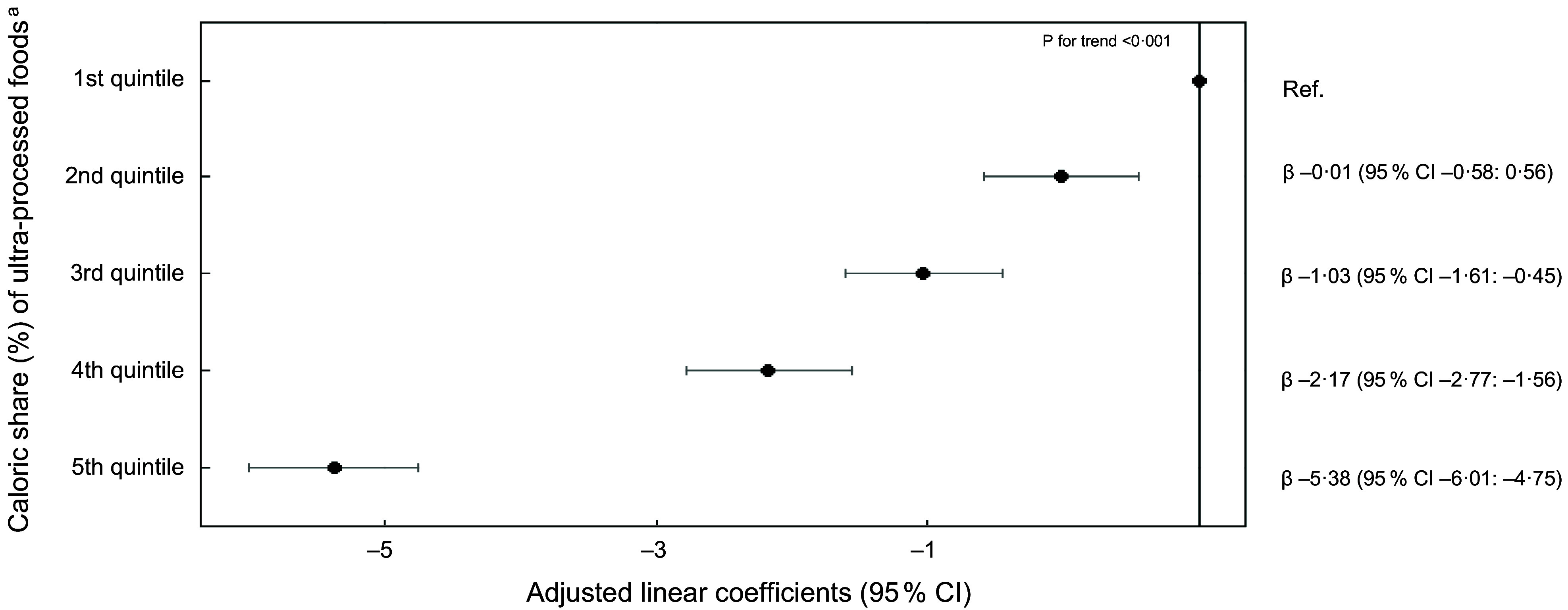
Adjusted linear coefficients and 95 % CI of association between Planetary Health Diet Index (PHDI) scores and quintiles of the caloric share (%) of ultra-processed foods (*n* 46 164). National Dietary Survey, 2017–2018. Legend: ^a^ 1^st^ quintile: mean (min – max): 0·43 (0 – 3·40); 2^nd^ quintile 6·83 (3·40 – 9·92); 3^rd^ quintile 13·3 (9·92 – 17·3); 4^th^ quintile 22·8 (17·3 – 29·6); 5^th^ quintile 45·1 (29·6 – 100). Model adjusted by sex, age, per capita income, self-reported race and residence area. Values are expressed as linear coefficients and 95 % CI

Crude and adjusted linear regression models showed a dose-response association between quintiles of ultra-processed food consumption and the PHDI total score (Figs. 2 and 3, respectively). The mean PHDI total score was 5·38 points lower (95 % CI –6·01, –4·75) in individuals in the highest quintile of consumption of ultra-processed foods, as compared to those in the first quintile (Fig. 3). Similarly, the mean PHDI total score was 1·31 points lower (95 % CI –1·98, –0·65) in individuals in the highest quintile of consumption of processed foods and 1·32 points lower (95 % CI –1·88, –0·76) in individuals in the highest quintile of consumption of culinary ingredients, as compared to those in the first quintile.

On the other hand, the mean PHDI total score was 8·34 points higher (95 % CI 7·76, 8·93) in individuals in the highest quintile of consumption of unprocessed or minimally processed foods, as compared to those in the first quintile. (Table 3).

**Table 3:** Linear regression coefficients and their 95 % CI for the association between the caloric share (%) of non-ultra-processed food groups (i.e. unprocessed or minimally processed foods, processed culinary ingredients and processed foods) and the Planetary Health Diet Index (PHDI) total score (*n* 46 164). National Dietary Survey 2017–2018

	Quintiles of caloric share (%) of unprocessed or minimally processed foods*									
	1^st^	2^nd^		3^rd^		4^th^		5^th^		*P* for trend
		Linear coefficients	95 % CI	Linear coefficients	95 % CI	Linear coefficients	95 % CI	Linear coefficients	95 % CI	
Model 1	ref.	3·69	3·05, 4·32	5·61	5·02, 6·19	6·88	6·28, 7·49	7·87	7·30, 8·45	<0·001
Model 2	ref.	3·60	2·97, 4·23	5·64	5·06, 6·22	7·00	6·39, 7·61	8·34	7·76, 8·93	<0·001
		Quintiles of caloric share (%) of processed culinary ingredients†								
	1^st^	2^nd^		3^rd^		4^th^		5^th^		
Model 1	ref.	1·23	0·56, 1·90	1·54	0·91, 2·16	0·71	0·08, 1·33	–1·03	–1·72, –0·35	<0·001
Model 2	ref.	1·42	0·77, 2·06	1·68	1·08, 2·29	0·74	0·13, 1·35	–1·31	–1·98, –0·65	<0·001
		Quintiles of caloric share (%) of processed foods‡								
	1^st^	2^nd^		3^rd^		4^th^		5^th^		
Model 1	ref.	0·71	–1·84, 3·25	0·48	–0·08, 1·04	0·85	0·34, 1·37	–1·17	–1·73, –0·61	0·070
Model 2	ref.	0·85	–1·67, 3·38	0·56	0·01, 1·10	0·74	0·24, 1·24	–1·32	–1·88, –0·76	0·017

* 1^st^ quintile: mean (min – max): 29.1 (0 – 39.7); 2^nd^ quintile 46.0 (39.7 – 51.5); 3^rd^ quintile 56.4 (51.5 – 61.3); 4^th^ quintile 66.6 (61.3 – 72.5); 5^th^ quintile 81.7 (72.5 – 100).

† 1^st^ quintile: 4.12 (0 – 6.95); 2^nd^ quintile 9.36 (6.96 – 11.7); 3^rd^ quintile 14.0 (11.7 – 16.4); 4^th^ quintile 19.5 (16.4 – 23.0); 5^th^ quintile 30.8 (23.0 – 100).

‡ 1^st^ quintile: 0 (0 – 0); 2^nd^ quintile 0.10 (0.02 – 0.16); 3^rd^ quintile 6.29 (0.16 – 11.5); 4^th^ quintile 15.9 (11.5 – 21.0); 5^th^ quintile 31.8 (21.0 – 100).

Model 1: unadjusted.

Model 2: adjusted by sex, age, per capita income, self-reported race and residence area. Values are expressed as linear coefficients and 95 % CI.

In the sensitivity analyses, the results from regression models employing Nova food groups in kcal showed consistent trends and directions similar to those obtained using % energy (see online supplementary material, Supplementary Table S3).

## Discussion

In this analysis of a nationally representative sample of the Brazilian population, we found that the consumption of ultra-processed foods was inversely associated with the adherence to a healthy and sustainable diet – proposed by the EAT-*Lancet* Commission. Similar associations were observed for the share of processed culinary ingredients and processed foods, while the consumption of unprocessed and minimally processed foods was directly associated with the PHDI scores.

Our results suggest that even though ultra-processed foods are not direct targets of the EAT-*Lancet* Commission recommendations, individuals with high adherence to its proposed diet also eat less of ultra-processed foods. This happens mainly because the PHDI adequacy components (fruits, vegetables, legumes, nuts and peanuts, and whole cereals) are usually eaten in the form of unprocessed or minimally processed foods (i.e. fresh or dry, unsugared and unsalted), especially in Brazil, where these foods comprise over 50 % of the typical diet^(23)^, leaving limited space for incorporating ultra-processed foods. In a recent meta-analysis with population-based studies from 13 countries (Australia, Brazil, Canada, Chile, Colombia, France, Italy, Korea, Mexico, Portugal, Taiwan, UK and US), Martini *et al.* ^(31)^ observed inverse linear relationships between the consumption of ultra-processed and unprocessed or minimally processed foods, suggesting that they may constitute opposite dietary patterns.

The group of ultra-processed foods has a poor nutrient profile characterised by lower amounts of protein, fibre and micronutrients, and higher contents of added sugar, total, saturated and trans-fatty acids^(31–33)^ (as compared to non-ultra-processed foods). Moreover, its consumption has been linked to diminished overall dietary quality, according to findings from nationally representative cross-sectional studies conducted in Brazil^(12)^, United States^(13)^ and Australia^(14)^. Conversely, previous results showed that higher scores in the PHDI (i.e. higher adherence to EAT-*Lancet* diet) were positively associated with higher intakes of vegetable proteins, polyunsaturated fats, fibres and micronutrients commonly present in fruits and vegetables, while negatively associated with animal protein, total fat, saturated fat, cholesterol and monounsaturated fat^(27,34)^. Alongside, the PHDI – that is, a higher adherence to EAT-*Lancet* sustainable diet – was also related with higher overall dietary quality^(27)^ as evaluated throughout the Revised-Brazilian Healthy Eating Index, a diet quality index adapted to the Brazilian context^(35)^.

The inverse association between adherence to the EAT-*Lancet* diet and ultra-processed foods can also be discussed from the perspective of environmental impacts. While greater adherence to the EAT-*Lancet* diet was associated with lower diet-related environmental impacts (e.g. carbon, water and land footprint) in modelling studies^(3,7)^, the consumption of ultra-processed foods has been also related to environmental impacts^(21,36,37)^, although still less explored. Garzillo *et al.* ^(36)^ using data from the 2007–2008 NDS in Brazil observed that ultra-processed foods were associated with higher carbon and water footprints, but this association remains only for water footprint, after controlling for age, sex, education, income and country regions. Silva *et al.* ^(21)^ evaluated the temporal trends of GHGE, water and ecological footprint of food purchases according to Nova food system between 1987 and 2018 using the Brazilian HBS and found that GHGE increased by 21 %, water footprint by 22 % and ecological footprint by 17 %, followed by the decrease in the share of unprocessed or minimally processed foods and an increase in the food purchase of processed and ultra-processed foods. In a national cross-sectional study with a representative French sample, Kesse-Guyot *et al.* ^(37)^ observed that those with the highest dietary share of ultra-processed foods also presented the highest diet-related environmental impacts indicators (e.g. GHGE, land use, use of fossil resources and ecological footprint); however, the association was fully mediated by the higher total energy intake associated with the consumption of ultra-processed foods.

Some studies have investigated the relationship of the EAT-*Lancet* diet with health outcomes, and the higher adherence to its recommendations was associated with lower cardiometabolic risk and lower odds for overweight and obesity in Brazilians^(38,39)^, lower risk of total mortality in Swedish population^(40)^ and lower risk of diabetes in UK citizens^(41)^ and in Mexican women^(42)^. In parallel to the benefits of the EAT-*Lancet* diet for human health, several other studies have addressed the impact of ultra-processed foods on the risk of noncommunicable diseases worldwide. Systematic reviews and meta-analysis of observational studies reported that ultra-processed foods were associated with increased risk of type 2 diabetes^(43)^, hypertension^(44)^, overweight/obesity^(45)^ and all-cause mortality^(19)^. Nilson *et al.*,^(46)^ in a modelling study, reported that the consumption of ultra-processed foods was associated with 22 % of premature deaths from cardiovascular diseases and 34 % of mortality attributed to all causes. Although the PHDI and the % energy intake from ultra-processed foods differ in their focus of interest (i.e. with PHDI evaluating adherence to a healthy and sustainable diet, while the Nova food system focus on classifying foods based on the degree of food processing and their impact on health), both systems have yielded significant results regarding diet-disease and diet-environmental impact associations.

Inverse associations between the adherence to EAT-*Lancet* diet and processed culinary ingredients and processed foods were also observed. According to Nova food system classification, these groups are, respectively, composed of oils, butter, salt, and sugar and industrialised products composed by adding a processed culinary ingredient to unprocessed or minimally processed foods. The EAT-*Lancet* recommends that food with high contents of sugar, sodium and fats should be consumed in moderation, due to the impact on human and planetary health^(8)^. According to the Dietary Guidelines for the Brazilian Population^(47)^, culinary ingredients and processed foods can be consumed moderately and be part of a healthy diet, especially when used in culinary preparations or recipes.

Our findings showed that ultra-processed foods comprise about 20 % of the calories of the Brazilian diet in 2017–2018, while unprocessed or minimally processed foods correspond to almost half of it. Despite that the Brazilian population is far from meeting the EAT-*Lancet* sustainable guidelines. Marchioni *et al.* ^(28)^ evaluated the adherence to EAT-*Lancet* diet throughout the PHDI in the 2017–2018 NDS and observed that the Brazilian population reached only 30 % of the possible points in the PHDI, indicating the poor adherence to the EAT-*Lancet* recommendations. Similar result was observed in a Brazilian cohort study, where adults reached only 40 % of the possible points in the PHDI^(27)^, even consuming more than 60 % of total calories from unprocessed or minimally processed foods and culinary ingredients^(48)^. A possible explanation to this may be the fact that EAT-*Lancet* strongly recommends moderation in the consumption of animal sources, even if they are unprocessed or minimally processed foods. As an example, the EAT-*Lancet* recommendation for red meat is 14 g/d, ranging from 0 to 28 g, and it is well known that Brazilians have a much higher consumption of red meat^(49)^. The 2017–2018 Brazilian HBS shows that the availability of both ultra-processed foods and red meat increased between 2002–2003 and 2017–2018, while the opposite was observed for plant-based unprocessed or minimally processed foods^(50)^.

We are familiar with the EAT-*Lancet* criticisms related to its dietary recommendations and the lack of discussion on ultra-processed foods^(9,10)^. Furthermore, although the report reinforces cultural adaptations to local realities can be proposed^(8)^, a global diet model may not be the best way to achieve sustainable food systems. According to Semba *et al.* ^(51)^, global adherence to the EAT-*Lancet* recommendations would have a 23 % reduction in GHGE; however, in low- and middle-income countries there would be an increase of up to 283 %. Accordingly, the adoption of the EAT-*Lancet* diet on a global scale would reduce about 12 % of the water footprint, but would increase the water footprint in 54 low- and middle-income countries^(52)^. In addition, Hirvonen *et al.* ^(53)^ analysis showed that the EAT-*Lancet* diet is not affordable for more than 40 % of the world’s population living in low- and middle-income countries. However, we recognise the role that such recommendations have played in recent years, strengthening and drawing attention to the debate on food systems and sustainable diets for human health within planetary boundaries^(10)^. In addition to proposing a global reference sustainable diet, the EAT-*Lancet* Commission also calls for more sustainable food production, by incorporating sustainable agriculture techniques and agrobiodiversity, such as soil regeneration, carbon sequestration, drip irrigation and soil water harvesting. Additionally, it advocates for reducing the consumption of animal foods, particularly beef, due to the burden it represents in terms of GHGE, as well as deforestation, and suggests shifting consumption towards plant-based foods with lower environmental impact, which can contribute to soil regeneration actions^(8)^.

Finally, our study has several strengths. We used the most recent nationally representative data on dietary intake, assessed throughout the 24-h dietary recall method, with the aid of a software developed exclusively for the survey, with data collected over a one-year period, which enabled capturing seasonal variations in dietary intake. Moreover, we used a previous validated diet index to assess the adherence to the EAT-*Lancet* diet^(27)^ and the most used system to classify ultra-processed foods^(11)^. However, our study also has limitations. Although the 24-hour dietary recall is often considered more accurate than the FFQ^(54)^, as it allows for the reporting of a wide variety of foods and beverages consumed the day before the interview, it still possesses certain limitations inherent to all methods for collecting dietary intake data, such as the memory bias^(54–56)^. Nonetheless, compared to the FFQ, the 24-hour dietary recall requires short-term memory, which implies less memory bias than the FFQ^(54–56)^. Moreover, in the NDS 2017–2018 study, the 24-hour dietary recall was applied by trained research interviewers who followed strict procedures to collect dietary intake, include using a software developed for this purpose and following the steps of the Automated Multiple-Pass Method^(57)^. Nonetheless, we used only the first 24-hour dietary recall, which means that our results are not of usual intake, although that one day is considered appropriate to estimate population average dietary intakes^(58)^.

Our study demonstrated the inverse relationship between the consumption of ultra-processed foods, processed foods and culinary processed ingredients with the adherence to a healthy and sustainable diet, while the share of unprocessed or minimally processed food in the total diet was directly associated with the adherence to sustainable dietary recommendations.

## Supporting information

Cacau et al. supplementary material Cacau et al. supplementary material

## Data Availability

The raw data used in this study are publicly available and can be downloaded from the following electronic address (https://www.ibge.gov.br/estatisticas/sociais/saude/24786-pesquisa-de-orcamentos-familiares-2.html?edition=28523&t=microdata). The analytic code of the PHDI computation will be made available upon request pending to the corresponding author.

## Supplementary material

For supplementary material accompanying this paper visit https://doi.org/10.1017/S1368980024001678
