# Supplementary material for: Adherence to the EAT-Lancet sustainable diet and ultra-processed food consumption: findings from a nationwide population-based study in Brazil
Source: Public Health Nutr. 2024 Oct 4;27(1):e183. doi: 10.1017/S1368980024001678 (PMC11505050; doi:10.1017/S1368980024001678)
Supplement: Cacau et al. supplementary material [file S1368980024001678sup001.docx]

| **Table S1**. Foods, beverages and ingredients included in the Planetary Health Diet Index (PHDI) components. National Dietary Survey, 2017-2018. | |
| --- | --- |
| PHDI components | Food and beverages |
| Nuts and peanuts | Nuts, almonds, peanuts, and seeds (such as sesame and chia seeds) |
| Legumes | Beans, chickpeas, lentils, peas, soy and soy products (tofu, soy milk and textured soy protein) |
| Fruits | All type of fruits, including dry fruits and coconut water. Include fruits used in juices, nectars and punches |
| Vegetables | All type of vegetables, excpet tubers |
| Whole cereals | Brown rice, whole bread, wheat bran, oatmeal and quinoa |
| Eggs | Chicken and other poultry eggs |
| Fish and seafood | Fish and seafood such as squid, shrimp, and crab. Including canned fish and seafood |
| Tubers and potatoes | Potatoes, sweet potatoes, yams, cassava and other types |
| Dairy | Cow and goat milks, yogurts, and cheeses. |
| Vegetable oils | Olive oils, margarine with or without salt, soybean oil, and sunflower oil. Include oils used in recipes |
| Dark green vegetables ratio | All dark green vegetables, such as broccoli, chicory, spinach, cabbage, malabar spinach, and *taioba* |
| Red-orange vegetables ratio | All red and orange vegetables, such as radish, beet root, squash, pumpkin, and tomato |
| Red meat | Beef, lamb and pork, including their processed meats (e.g., sausage, ham and salami). |
| Chicken and substitutes | Chicken and other poultry, including their processed. Includes intakes exceeding the upper limit of eggs and/or fish and seafood |
| Animal fats | Lard, tallow, butter and other dairy fats (e.g., sour cream and cheese cream) |
| Added sugars | Table white or brown sugar and honey used as ingredients in processed or culinary products and the added sugar to manufactured foods and beverages |

| **Table S2**. Distribution (%) of the total daily energy intake according to Nova food groups. Brazilian National Dietary Survey, 2017-2018. | | | |
| --- | --- | --- | --- |
|  | **Mean** | **95% CI** | |
| **Unprocessed or minimally processed foods** | **53.4** | **53.0** | **53.8** |
| Rice | 10.9 | 10.7 | 11.1 |
| Red meat | 7.4 | 7.1 | 7.6 |
| Beans | 6.6 | 6.5 | 6.8 |
| Chicken | 5.4 | 5.3 | 5.6 |
| Fruit | 3.1 | 3.0 | 3.2 |
| Milk | 2.8 | 2.7 | 2.9 |
| Pasta | 2.5 | 2.3 | 2.6 |
| Vegetables | 1.9 | 1.8 | 1.9 |
| Pork | 1.8 | 1.7 | 1.9 |
| Roots and tubers | 1.8 | 1.7 | 1.9 |
| Fruit juice | 1.6 | 1.5 | 1.7 |
| Eggs | 1.4 | 1.4 | 1.5 |
| Cassava flour | 1.4 | 1.3 | 1.5 |
| Fish | 1.1 | 1.0 | 1.2 |
| Other cereals | 0.8 | 0.8 | 0.9 |
| Coffee and tea | 0.7 | 0.7 | 0.7 |
| Wheat flour | 0.5 | 0.5 | 0.5 |
| Other flours | 0.4 | 0.3 | 0.4 |
| Offal | 0.3 | 0.2 | 0.3 |
| Nuts | 0.2 | 0.1 | 0.2 |
| Other vegetables | 0.2 | 0.1 | 0.2 |
| Other unprocessed and minimally processed foods | 0.3 | 0.2 | 0.3 |
| **Processed culinary ingredients** | **15.6** | **15.4** | **15.8** |
| Olive oil | 7.7 | 7.6 | 7.9 |
| Sugar | 5.8 | 5.7 | 5.9 |
| Butter | 1.0 | 0.9 | 1.0 |
| Other processed culinary ingredients | 0.8 | 0.7 | 0.8 |
| Lard | 0.3 | 0.3 | 0.4 |
| **Processed foods** | **11.3** | **11.1** | **11.5** |
| Bread | 8.2 | 7.9 | 8.4 |
| Processed cheese | 1.6 | 1.5 | 1.7 |
| Fermented alcoholic beverages | 0.7 | 0.6 | 0.8 |
| Salted meat | 0.4 | 0.4 | 0.5 |
| Fruit sweets | 0.2 | 0.2 | 0.3 |
| Other processed foods | 0.2 | 0.2 | 0.2 |
| **Ultra-processed foods** | **19.7** | **19.3** | **20.1** |
| Margarine | 2.8 | 2.7 | 2.8 |
| Salted snacks | 2.5 | 2.4 | 2.6 |
| Ultra-processed breads | 2.1 | 2.0 | 2.2 |
| Sweet snacks | 1.7 | 1.6 | 1.8 |
| Sausages | 1.6 | 1.5 | 1.7 |
| Ice-cream | 1.4 | 1.3 | 1.5 |
| Soda | 1.3 | 1.2 | 1.4 |
| Fast food | 1.1 | 1.0 | 1.2 |
| Dairy drinks | 1.1 | 1.0 | 1.1 |
| Pizza | 0.9 | 0.6 | 1.3 |
| Savories | 0.7 | 0.6 | 0.8 |
| Other beverages | 0.6 | 0.6 | 0.7 |
| Ready to eat | 0.6 | 0.5 | 0.7 |
| Sweet cream | 0.4 | 0.3 | 0.4 |
| Milk cream | 0.4 | 0.4 | 0.4 |
| Other ultra-processed foods | 0.4 | 0.4 | 0.5 |
| Fruit sweets are typical preparations in Brazil, made by combining fruits and sugar, such as fruit jam. Others mean foods that are less commonly consumed by the Brazilian population. | | | |

| **Table S3**. Adjusted linear regression coefficients and their 95% CI for the association between the calories (kcal) of Nova food groups and the Planetary Health Diet Index (PHDI) total score (*n* 46,164). National Dietary Survey 2017-2018. | | | | | | |
| --- | --- | --- | --- | --- | --- | --- |
|  | Quintiles of calories (kcal) | | | | |  |
|  | 1^st^ | 2^nd^ | 3^rd^ | 4^th^ | 5^th^ | p for trend |
| Unprocessed or minimally processed foods | ref. | 3.23 (2.64: 3.82) | 4.37 (3.77: 4.98) | 5.03 (4.37: 5.70) | 5.26 (4.61: 5.92) | <0.001 |
| Processed culinary ingredients | ref. | 1.08 (0.41: 1.75) | 0.44 (–0.23: 1.12) | –0.14 (–0.82: 0.54) | –1.17 (–1.89: –0.45) | <0.001 |
| Processed foods | ref. | –1.12 (–3.89: 1.65) | 0.76 (0.29: 1.23) | –0.38 (–1.10: 0.35) | –0.88 (–1.44: –0.33) | 0.014 |
| Ultra-processed foods | ref. | –0.23 (–0.79: 0.34) | –1.26 (–1.87: –0.65) | –2.39 (–3.01: –1.77) | –5.18 (–5.79: –4.57) | <0.001 |
